# From PERFORM to PERFORM2Scale: lessons from scaling-up a health management strengthening intervention to support Universal Health Coverage in three African countries

**DOI:** 10.1093/heapol/czae063

**Published:** 2024-07-09

**Authors:** Joanna Raven, Wesam Mansour, Moses Aikins, Susan Bulthuis, Kingsley Chikaphupha, Marjolein Dieleman, Maryse Kok, Tim Martineau, Freddie Ssengooba, Kaspar Wyss, Frédérique Vallières

**Affiliations:** Department of International Public Health, Liverpool School of Tropical Medicine, Pembroke place, Liverpool L3 5QA, UK; International Public Health, Liverpool School of Tropical Medicine, Pembroke Place, Liverpool L3 5QA, United Kingdom; School of Public Health, College of Health Sciences, University of Ghana, PO Box LG13, Legon, Accra, Ghana; Royal Tropical Institute, Mauritskade 63, 1092 AD, Amsterdam, Netherlands; Reach Trust, PO Box 1597, Lilongwe, Malawi; Royal Tropical Institute, Mauritskade 63, 1092 AD, Amsterdam, Netherlands; Royal Tropical Institute, Mauritskade 63, 1092 AD, Amsterdam, Netherlands; International Public Health, Liverpool School of Tropical Medicine, Pembroke Place, Liverpool L3 5QA, United Kingdom; Department of Health Policy and Planning, School of Public Health, Makerere University, PO Box 7072, Kampala, Uganda; Swiss Tropical and Public Health Institute & University of Basel, Kreuzstrasse 2, 4123 Allschwil, Basel, Switzerland; Trinity Centre for Global Health, Trinity College Dublin, 7-9 Leinster Street South, Dublin D02 K104, Ireland

**Keywords:** Scale-up, management strengthening, district health management team

## Abstract

Strengthening management and leadership competencies among district and local health managers has emerged as a common approach for health systems strengthening and to achieve Universal Health Coverage (UHC). While the literature is rich with localized examples of initiatives that aim to strengthen the capacity of district or local health managers, particularly in sub-Saharan Africa, considerably less attention is paid to the science of ‘how’ to scale-up these initiatives. The aim of this paper is thus to examine the ‘process’ of scaling-up a management strengthening intervention (MSI) and identify new knowledge and key lessons learned that can be used to inform the scale-up process of other complex health interventions, in support of UHC. Qualitative methods were used to identify lessons learned from scaling-up the MSI in Ghana, Malawi and Uganda. We conducted 14 interviews with district health management team (DHMT) members, three scale-up assessments with 20 scale-up stakeholders, and three reflection discussions with 11 research team members. We also kept records of activities throughout MSI and scale-up implementation. Data were recorded, transcribed and analysed against the Theory of Change to identify both scale-up outcomes and the factors affecting these outcomes. The MSI was ultimately scaled-up across 27 districts. Repeated MSI cycles over time were found to foster greater feelings of autonomy among DHMTs to address longstanding local problems, a more innovative use of existing resources without relying on additional funding and improved teamwork. The use of ‘resource teams’ and the emergence of MSI ‘champions’ were instrumental in supporting scale-up efforts. Challenges to the sustainability of the MSI include limited government buy-in and lack of sustained financial investment.

Key messages

- PERFORM2Scale successfully scaled-up a management strengthening intervention to 27 districts across 3 countries, and integrated the intervention into policies and routine practice.
- Working with champions who are well-positioned within existing political structures and who can readily identify current practices and policies that would benefit from the integration of an intervention is critical.
- Considering how interventions might be better communicated and marketed to suit existing priorities and policies is essential, even if this may result in substantially modified, albeit more contextually appropriate, interventions.
- The importance of securing funding commitment for whatever form the intervention eventually takes is needed to ensure its longer-term sustainability.

## Introduction

Strong management and effective leadership at all levels of the health system are critical to ensuring equitable access to quality health care for communities (Daire *et al*., 2014; Meessen and Malanda, 2014). Accordingly, and particularly within decentralized health systems, strengthening management and leadership competencies among district and local health managers has emerged as a common approach for health systems strengthening and to achieve Universal Health Coverage (UHC). For example, the ‘Maternal and Newborn Health in Ethiopia Partnership’ programme adapted a collaborative improvement strategy to develop leadership capacity to improve community maternal and neonatal health (Stover *et al*., 2014). Likewise, Tetui *et al*. (2017) employed participatory action research (AR) to strengthen health managers’ capacity in Eastern Uganda. Common to these interventions is the use of collaborative approaches empowering district-level health managers, who, given knowledge of their context’s unique challenges, are arguably better placed to independently identify areas of improvement; derive and prioritize locally appropriate, feasible solutions; and subsequently monitor and evaluate the success of these solutions (Kwamie *et al*., 2015; Waiswa *et al*., 2021; Bosongo *et al*., 2023).

Employing a similar approach, the PERFORM project, which ran from 2011 to 2015, employed the Management Strengthening Intervention (MSI) to effectively strengthen management competencies of district health management team (DHMT) members in Ghana, Tanzania and Uganda (Martineau *et al*., 2018).

### The MSI

**Figure 1.**
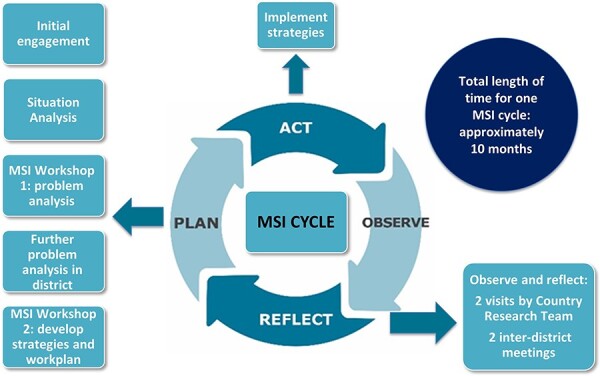
Management strengthening intervention

The MSI uses an AR approach to enable DHMTs to: analyse their own workforce performance and service delivery problems and develop appropriate workplans (plan), implement the workplans (act), and then learn about what works and does not work to address the problems (observe and reflect) (see Figure 1). DHMTs are encouraged to use existing government budget and local donor funds to support implementation of the strategies.

In the ‘act’ stage, the DHMTs implement the strategies over a period of approximately 8 months. The observation and reflection stages happen concurrently throughout implementation.

In the ‘observation’ stage, the DHMTs observe and document how each of the strategies are being implemented. They can use the indicators developed in the workplan to monitor the effects of the strategies.

The ‘reflection’ stage is when the DHMTs can take stock of whether, and to what extent, problems have been resolved and why. If a DHMT finds that one of the strategies they are implementing is not working, they can modify or drop the strategy and add other strategies. Inter-district meetings and research teams’ support visits facilitate this process.

The DHMTs now move into MSI cycle 2, either continuing to work with the strategies as they have been effective; adapting the strategies; or addressing another problem identified in the situation analysis. The PERFORM2Scale project funded DHMT, research team and other stakeholders’ attendance at the workshops and inter-district meetings, but not funding for implementation of the strategies.

Guided by a toolkit (PERFORM2Scale, 2022), research teams can adapt the MSI to suit local needs in terms of timing, duration of cycles, number of workshops and support meetings and monitoring/tracking mechanisms. In addition, the MSI adopts the following principles:

- DHMTs must choose and prioritize which problem(s) to address.
- Strategies to address the locally selected problems must assume that no additional resources are available for implementation.
- Strategies should not be so ambitious that they risk becoming unfeasible.
- The MSI is implemented as a team.
- Experiences and learnings are regularly shared with other district teams facing the same challenges.

### Scaling-up the MSI

While there is some evidence to suggest that leadership and MSIs are associated with improved service delivery (Tetui *et al*., 2017; Martineau *et al*., 2018), less understood is ‘how’ to scale and sustain these innovations over time. More specifically, and whereas previous research has identified determinants of successful scale-up (Bennett *et al*., 2017; Ghiron *et al*., 2021; Bulthuis *et al*., 2023), the outcomes of scale-up have yet to be linked to its process.

PERFORM2Scale project sought to scale-up PERFORM’s MSI across a minimum of 27 districts in Ghana, Uganda and Malawi from 2017 to 2022. In doing so, we generated new knowledge and key lessons learned that can be used to inform the scale-up process of other complex health interventions going forward.

### The scale-up approach

**Figure 2.**
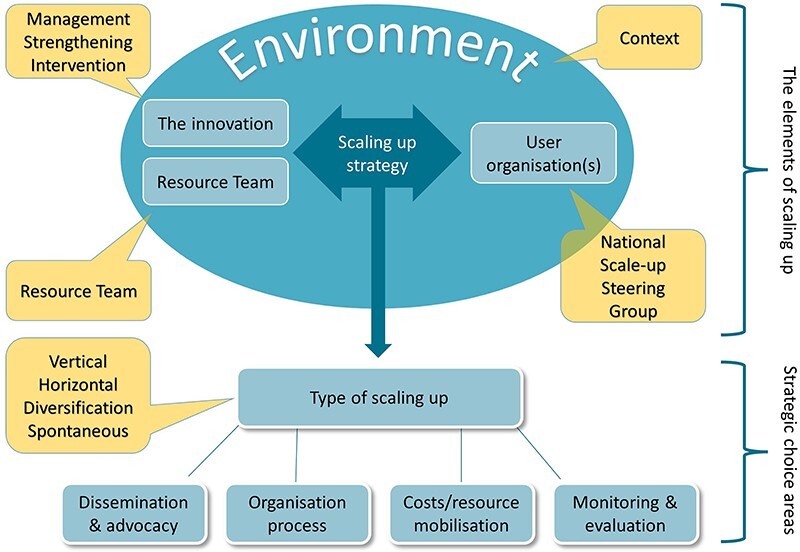
Scale-up approach

PERFORM2Scale project adapted the systematic approach for scale-up developed by ExpandNet and the World Health Organization (WHO), which had been previously tested in different contexts (World Health Organization /ExpandNet, 2010) (Figure 2). This approach was selected as it uses both a ‘horizontal’ scale-up approach (i.e. ‘expansion and/or replication of the intervention across the country’) and a ‘vertical’ scale-up approach (i.e. institutionalization through policy, political, legal, budgetary or other health systems changes to support the horizontal scale-up) to achieve an overall sustainable scale-up process. Scale-up is thus guided by a strategy which brings together horizontal and vertical scale-up approaches as well as ensuring sustainability. In each country, we also identified ‘user organizations’ that would adopt and widen the scale-up process following the completion of PERFORM2Scale project. User organizations included Ministries of Health (MoH) in all countries, as well as the Ministry of Local Government and Rural Development (MoLGRD) in Malawi and the Ghana Health Service (GHS) in Ghana. From these user organizations, a structure—termed the National Scale-up Steering Group (NSSG)—was established in each country to support and eventually lead on the scale-up process in each country (handover of the MSI and scale up to this group). The role of the NSSG was designed to develop each country’s initial scale-up strategy, identify participating district groups, review progress on the scale-up at regular intervals and, where necessary, revise the scale-up strategy accordingly, as well as develop funding plans for further scale-up beyond the end of the project, absorbing the MSI into country policies and guidelines.

The scale-up process was designed to start with one group of three neighbouring districts (District Group 1) to implement the first MSI cycle. Following the completion of the first cycle (10–12 months), a second MSI cycle was planned for District Group 1 to continue the management strengthening process, whilst a first MSI cycle was initiated in a second group of three neighbouring districts (District Group 2). In this way, the district strengthening process is sustained (and further embedded) in the District Group 1 and horizontal scale-up is achieved via the geographical spread of districts using the MSI cycle. Resource Team (RT) members in each country, comprised of a mix of district, regional and national health and local government officials, supported the implementation for the MSI cycles as part of this scale-up approach. Country-based and European research teams jointly studied the scale-up process. See Table 1 for project structures, roles and support provided.

**Table 1.** PERFORM2Scale project structures established to support scale-up

Structure	Roles	Support provided
National Scale Steering Group (NSSG)	Develop initial scale-up strategy Identify participating district groups Review progress on the scale-up and revise the scale-up strategy Develop funding plans for further scale-up beyond the end of the project	Travel and allowance for attendance at meetings and workshops
Resource Team (RT)	Support the implementation of the MSI in the districts Support NSSG to develop the scale-up plan	Travel and allowance for attendance at meetings and workshops
Research team includes country-based researchers (from Ghana, Malawi and Uganda) and European researchers (from UK, Ireland, Netherlands and Switzerland)	Support the implementation of the MSI in the districts, alongside the RT, reducing their support as RT becomes more confident Support the development of the scale-up strategy Conduct process and outcome evaluation research	Salary support Travel for meetings and workshops

The aim of this paper is to examine the process of scaling-up the MSI and identify new knowledge and key lessons learned that can be used to inform the scale-up process of other complex health interventions.

## Methods

### Study settings

Ghana, Malawi and Uganda were selected as study countries in PERFORM2Scale project as all three countries have decentralized management of health services to the district level (Aikins *et al*., 2018; Kwalamasa *et al*., 2018; Ssengooba *et al*., 2018). DHMTs are thus more likely to have the ‘decision space’ to address local challenges (Bossert and Beauvais, 2002). While Uganda and Ghana have a more established decentralized structure, Malawi—which initiated a process of health sector decentralization in 2004—has yet to fully decentralize the management of human resources (Bulthuis *et al*., 2021). Further, all three countries face challenges in achieving UHC, experience critical shortages of healthcare workers and have clear and ambitious policies and plans to improve their population’s health outcomes (World Health Organization, 2016).

### Data collection

The qualitative methods used to identify lessons about scaling-up the MSI are summarized in Table 2. Data were collected in 2021 and tools are available in Supplementary File 1. We also documented all activities throughout MSI and scale-up implementation. Specifically, the following methods were used:

- In-depth interviews with DHMTs: interviews were held in four districts (two districts from district group 1 and two districts from district group 2) in Ghana and Malawi, and all six districts from districts groups 1 and 2 in Uganda. DHMT members were purposefully sampled based on their engagement with the horizontal scale-up. Selection of DHMT members took into account their functions, type and level of involvement, gender and seniority. In Uganda and Malawi, members of the district council were included as they were involved in the intervention, which was not the case in Ghana. In total, 39 interviews were conducted: 11 in Ghana, 12 in Malawi and 16 in Uganda. The interviews focused on experiences and effects of the MSI, horizontal scale-up process and factors affecting the horizontal scale-up. Interviews took between 60 and 90 minutes, and were conducted by European and country research team members.
- Scale-up assessment: the purpose of this assessment was to explore the scale-up process including the facilitating and hindering factors, from the perspective of national stakeholders. In Ghana, three NSSG members and five RT members participated. In Malawi, two NSSG members, five RT members and three additional stakeholders who were knowledgeable about the scale-up process were included. In Uganda, one RT member and one NSSG member participated. Participants first individually scored statements about the scale-up. These statements were informed by a literature review that identified barriers and facilitators to scale-up of public health interventions (Bulthuis *et al*., 2020). The research team then facilitated a discussion of the scores using a topic guide. The group discussions took about 90 minutes.
- Reflections with research teams: during these group discussions, research team members from other countries supported the research team members to reflect on the implementation of the intervention and its scale-up and factors of influence in their country. For each country, three to four research team members participated in the group reflections. The research team members used an interview guide, which was based on the scale-up assessment interview guide. These sessions facilitated deeper reflection of the findings from the in-depth interviews and scale-up assessment. The reflective sessions took about 90 minutes.
- Tracking MSI and scale-up implementation: all activities of the implementation of the MSI in each district and the scale-up process in each country were recorded in a tracking excel tool. These included, e.g. timing and participation of workshops, visits to the districts, NSSG meetings and RT meetings.

**Table 2.** Overview of methods and participants

Method	Country	Number of participants
**In-depth interviews**	Ghana	4 districts (DG1 and DG2): 11 DHMT members (4 F; 7 M)
	Malawi	4 districts (DG1 and DG2): 11 DHMT members and 1 District Council (4 F; 8 M)
	Uganda	6 districts (DG1 and DG2): 15 DHMT members and 1 HR officer (6 F; 10 M)
**Scale-up assessment**	Ghana	3 NSSG members (1 F; 2 M); 5 RT members (2 F; 3 M)
	Malawi	2 NSSG members (1 F; 1 M); 5 RT members (3 F; 2 M) Additional stakeholders: 1 government, 1 UN organization and 1 bilateral donor (1 F; 2 M)
	Uganda	1 RT member and 1 NSSG member (2 M)
**Country research team reflection**	Ghana	4 research team members (1 F; 3 M)
	Malawi	4 research team members (4 M)
	Uganda	3 research team members (2 F; 1 M)

DG = district group.

### Data management, analysis and synthesis

Qualitative data were analysed using a thematic framework analysis approach which facilitates rigorous and transparent analysis (Ritchie *et al*., 2003). First, recorded interviews and group discussions were transcribed verbatim and read thoroughly for the purpose of familiarization. We then developed a coding framework based on reading of the transcripts and the PERFORM2Scale project Theory of Change (see Supplementary File 2). This coding framework was then applied to all the transcripts. Data were extracted to Microsoft Excel tables under each data extraction heading, as per our Theory of Change (see Table 3). From these tables, we identified scale-up outcomes and the factors affecting these outcomes. These were then discussed during a consortium workshop held in February 2022 and further developed into narratives refined by the whole consortium after reviewing for accuracy and coherence.

**Table 3.** Data extraction headings as per Theory of Change

Horizontal scale-up pathway—MSI	Vertical scale-up pathway
Adaptations of the MSI	User organizations convinced of the value of MSI (based on PERFORM and other examples)
DHMTs capacitated in MSI approach	Scale-up infrastructure developed a) NSSG b) Resource Team c) scale-up strategy
Management skills, team confidence and independence increased, and teamwork strengthened	Champions emerged who support and advocate for MSI scale-up
Workplans developed by DHMTs are feasible and address real problems	Wider group of stakeholders convinced of the value of MSI scale-up
Selected workforce performance and service delivery problems addressed	National/regional resource allocation and scale-up infrastructure support existing MSI cycles and ongoing scale-up
New management cycles conducted and new DHMTs included in programme	Health polices and plans include MSI
MSI embedded in DHMT working method without external inputs	Expertise for scaling up is applied to other systems areas
Improved general management and workforce performance management at district level	Improved service delivery and UHC
Improved workforce performance	

For the quantitative data, the implementation of the MSI was tabulated by country and year and cycle number.

## Results

The results are presented under five scale-up outcomes: (1) scale-up structures established; (2) horizontal scale-up successful; (3) management strengthened when MSI scaled-up; (4) country scale-up strategies developed; and (5) some integration of MSI into policies and practice in Uganda and Malawi, but no financial support. In Ghana, there is currently no evidence of political and financial support for the scale-up of the MSI. These outcomes, together with the various factors affecting each of these outcomes, are summarized in Table 4.

**Table 4.** Summary of key findings

	Outcomes	Factors affecting outcomes
**1a**	Scale-up structures established and functioning: NSSGs	High workload of NSSG members NSSG viewed as parallel structure Power dynamics affected functioning Alternative approaches employed when NSSGs not functioning
**1b**	Scale-up structures established and functioning: RTs	Stability of the RT Expert knowledge and seniority of RT Research team support to facilitate MSI
**2**	Horizontal scale-up was achieved	Plan for horizontal scale-up Support for MSI facilitation Locally appropriate adaptations to the MSI COVID-19—priority given to pandemic control
**3**	Management strengthened when MSI scaled-up	Multiple MSI cycles Research team role
**4**	Country scale-up strategies were developed	Vision and clarity Loss of momentum with COVID-19 emergence
**5**	Some integration of MSI into policies and practice in Uganda and Malawi, but no financial support	Champions for scaling-up the MSI Generating and sharing robust quantitative and costing data early Power and politics—aligning with people with power and with political interests

### 1a: scale-up structures established: NSSG

NSSGs were established in 2017 and 2018, following stakeholder analyses and engagement activities in each country. Table 5 offers a summary of the individuals from different government ministries and departments and other organizations who were identified as having an interest and influence to scale-up the MSI, to act as potential members of the NSSG. To support the NSSGs, indicative functions of the NSSG were outlined in the PERFORM2Scale project scale-up guidelines and communicated to the NSSG. The NSSGs supported the initial plan for horizontal scale-up; however, they did not take a leading role in other functions, in particular the development of funded plans for further scale-up after PERFORM2Scale project’s completion.

**Table 5.** NSSG and RT members

	NSSG	RT
**Ghana**	Senior leader, Ghana Health Services Regional Director of Health Services, Ghana Health Services Human Resource Directorate, Ghana Health Services Health Research and Development Division, Ghana Health Services Policy, Planning, Monitoring and Evaluation Division, MoH Senior leader, Christian Health Association of Ghana	Regional Director of Health Services, Ghana Health Services Research Officer, Regional Administration, Ghana Health Service Public Health Officer Regional Office, Ghana Health Service DHMT members
**Malawi**	Senior leader, Planning and Development, MoH Senior leader, Human Resource Management, MoH Senior leader, Clinical Services, MoH Senior leader, Quality Management, MoH Senior leader, Human Resources, MoLGRD	Leader, Quality Management, MoH Two Quality Management Officers, MoH Leader, Clinical services, MoH Leader, Human Resource Management, MoH Human Resource Management Officer, MoH District Health Officer Leader, Expanded Programme of Immunisation
**Uganda**	Senior leader, MoH Senior leader, Quality Assurance and Improvement Department, MoH Senior leader, Human Resource Management Department, MoH Senior leader, Planning Department, MoH	Senior leader for nursing and midwifery, MoH Senior leader for Human Resource Management, MoH Leader, Quality Assurance and Improvement Department, MoH Human Resource Officer, MoH

Establishing the NSSG in Malawi was delayed, partly because the MSI was a new intervention, but also because it took time to agree whether the scale-up process should be led by the MoH or the MoLGRD.

#### Factors affecting the functioning of the NSSGs

##### High workload of NSSG members

As high-level government officials with competing priorities, scheduling and meeting attendance emerged as a common challenge across all three countries. Alternative approaches are described below.

##### NSSG viewed as a parallel structure

In all countries, the NSSGs were viewed as either a parallel structure (Uganda) or as an unofficial structure developed by the PERFORM2Scale project, rather than owned by the MoH. Consequently, issues arose in that the NSSG did not have clear reporting mechanisms to senior management within the MoH (Ghana).

##### Power dynamics

The Malawi NSSG, once established, faced numerous challenges linked to inter- and intra-departmental power dynamics, which limited the degree to which the NSSG and RT could work together effectively, and, in turn, the degree to which scale-up was facilitated. These challenges included continued discussions about which government department should manage the scale-up process, and frequent transfer of NSSG members to different departments resulting in new members of NSSG and a loss of institutional memory of PERFORM2Scale at the leadership level. Consequently, the RT took on most of the role of the NSSG, including planning for vertical scale-up.

##### Alternative approaches

In Uganda, the limited functioning of the NSSG was addressed by appointing a focal person (the NSSG-FP) who became pivotal to all scale-up processes. The NSSG-FP engaged MoH Technical Working Groups who then provided technical and stakeholder guidance to PERFORM2Scale project. However, the forum for discussing and planning the scale-up strategy was limited, with most discussions taking place with only the NSSG-FP. The NSSG-FP also served as the primary gatekeeper to the Quality Improvement Department in the MoH. In Ghana, frequent turnover of NSSG members contributed to the limited functioning of the NSSG. Consequently, a Regional Scale-up Steering Group (RSSG) was established in addition to the NSSG, as regional actors were seen as more engaged and could more readily relay information to the NSSG:

If we have, for example, a district director or a regional programme officer or director, who is actively part of the RSSG who has implemented an MSI programme that has yielded good results, promoted to national or another region, it becomes easier for this person to also set up a team and continue with implementing the MSI process in that region or position. (Research Team, Ghana)

### 1b: scale-up structures established: RTs

RTs were established in each country by 2018. Team composition varied across the three countries, with the involvement of district, regional and national members (Table 4). In Ghana, the RT was composed of selected regional health directorate members and DHMT members who had gone through the MSI in District Group 1. This facilitated better relationships between the regional directorate and the district, and improved facilitation of the MSI needed for scale-up. In Malawi, regional and national members from MoH and MoLGRD were also included, with some members added during the implementation of the MSI. In Uganda, the RT was composed of national-level actors who were lower-ranking officers from MoH.

The RTs worked well in all countries, co-facilitating MSI activities and supporting expansion to new districts. They also championed the MSI amongst stakeholders and played a part in developing the scale-up strategy and in some cases, the integration of the MSI into policy.

#### Factors affecting functioning of the RT

##### Stability of the RT

In all three countries, the RT was more stable than the NSSG, with low turnover of members resulting in stronger institutional memory of the MSI and scale-up. The members generally held lower ranking roles than members of the NSSG and were therefore more accessible and available to implement the MSI.

##### Expert knowledge and seniority of RT members

RT members were well-respected in all countries and therefore provided legitimacy to the MSI. In Uganda, RT members were actively engaged in MSI activities in the districts, and provided expertise on technical issues, such as health workforce performance, which the DHMTs valued greatly. However, they were ultimately too busy to take over the day-to-day facilitation of the MSI in the districts. Nevertheless, their seniority enabled the facilitation of the integration of the MSI into the Quality Improvement framework, thus playing a critical role in the vertical scale-up.

##### Research team support to facilitate the MSI

The research teams provided guidance through meetings and documentation, and facilitated the MSI alongside the RTs. In Malawi, responsibility for organizing and facilitating the MSI workshops was eventually handed over to the RT, who became the ‘face of the project’. In Ghana, the RT members gained the knowledge and skills to implement the MSI in other districts and sub-districts:

The trainings we have had with them [research team], the visits that they have paid to us and a lot of activities that we have also carried out. These have all made us more confident […]. Now we can take people through the MSI cycle, how to prioritize your problems, the matrix used to prioritize your problems, and we can also share our experiences with them that we were able to yield results without any external resources. (RT member, Ghana)

### 2: Horizontal scale-up was achieved

Table 6 summarizes when each MSI cycle was implemented across a total of 27 districts, represented within three district groups in each country. The geographical location of each district is available in the individual country maps available in Supplementary File 3.

**Table 6.** Numbers of districts and MSI cycles in Ghana, Uganda and Malawi

District group/country	Implementation year				
Year	2018	2019	2020	2021	# Districts
**Ghana**					
**DG1**	Cycle 1	Cycle 2	Cycle 2 cont’d	Cycle 2 cont’d	3
**DG2**		Cycle 1	Cycle 1 cont’d	Cycle 2	3
**DG3**				Cycle 1	3
**Uganda**					
**DG1**	Cycle 1	Cycle 2	Cycle 3 cont’d	Cycle 3 cont’d	3
**DG2**		Cycle 1	Cycle 2	Cycle 2 cont’d	3
**DG3**			Cycle 1	Cycle 1 cont’d	3
**Malawi**					
**DG1**	Cycle 1	Cycle 2	Cycle 2 cont’d	Cycle 2 cont’d	3
**DG2**		Cycle 1	Cycle 1 cont’d	Cycle 1 cont’d	3
**DG3**				Cycle 1	3
**Cycles**	**3**	**6**	**7**	**9**	**27**

DG = district group.

#### Factors affecting the horizontal scale-up

##### Plans for horizontal scale-up

Plans for horizontal scale-up were developed by the NSSG and RT in each country and included selection of districts, introduction of PERFORM2Scale project to these districts and a corresponding timeline. These plans were viewed as instrumental to facilitate the MSI across these districts.

##### Support for MSI facilitation

A detailed toolkit (PERFORM2Scale, 2022) including guidance on the workshops, guidelines on developing health workforce and health systems strategies, presentations and reporting templates was available. A face-to-face training workshop with research teams was held in Uganda in 2018 to go through the MSI process, cascaded to the RTs through meetings. Regular webinars were also held to discuss experiences, challenges and solutions whilst implementing the MSI. In all countries, the research team played a pivotal role in facilitating the MSI and supporting the RT in taking on this role (see section 1b).

##### MSI adaptations

The research teams and RTs introduced locally appropriate improvements to the MSI, based on their experiences with multiple district groups and cycles. More time for relationship building with the DHMTs was needed: the Ugandan research team added a pre-visit before the formal orientation, additional ongoing support through supplementary visits to the districts were provided and the Malawian research team extended the second workshop from 2.5 to 3.5 days. Moreover, and as research teams gained a better understanding of how health service decisions were made at district level, other actors from local government were involved in the MSI in addition to the DHMT:

So, when we come with the project plan, the first activity we do is disseminate that project plan and we call all the in-charges, the politicians, and we inform them about the plan and what we intend to do. So, from the beginning, all the key district stakeholders are brought on board. (DHMT member, Uganda)

While implementation work plans were originally scheduled over an 8-month period (partly so that we could allow for the completion of three completed cycles in district group 1), the implementation period was ultimately extended to align with the districts’ annual planning cycles.

##### COVID-19

The major hindrance to the horizontal scale-up was the COVID-19 pandemic in March 2020. Specifically, social distancing measures meant that the planned MSI support activities (workshops, visits, etc.) could no longer take place and the attention of the DHMTs was diverted from the implementation of their workplans. However, many of the participating DHMTs picked up the momentum for the MSI before the end of the project by implementing and monitoring their workplans and actively participating in workshops and research teams’ visits, suggesting that the approach was sufficiently embedded in their way of working. This was further demonstrated by the application of the MSI to COVID-19-related challenges in Uganda. As one DHMT member from Uganda described:

I’m able to apply these principles in the day-to-day activities. […] now we are […] grappling with COVID-19, […]no one was prepared, […] we lacked resources to go to the communities and do case tracing and what have you, but we had to use the available resources to respond to the problem […]. And that is basically the principle of MSI which we applied.

### 3: Management competencies strengthened when scaling up

#### Improved confidence and independence

DHMTs strengthened their problem analysis and solving skills and were able to develop feasible and logical strategies and accompanying workplans. Through the MSI process, they were more able to address problems hampering their ability to meet their service delivery targets more effectively and efficiently. Although the process of problem-solving was not completely new to most DHMTs, taking responsibility for problem selection, strategy design and the implementation was somewhat novel. This was not seen as extra work but as a different approach to their existing role and responsibilities. Some DHMTs demonstrated increased independence by addressing problems that they would have otherwise deferred until they received regional or national support:

I have learned a lot because through the MSI we have been able to handle a lot of our problems at our level. We at the district level have been able to solve some of our issues locally, by not having to wait for the region or national level. (DHMT member, Uganda)

#### Improved efficiency

DHMTs reported that the MSI encouraged them to think more innovatively about the use of existing resources. Some DHMTs reported that they saw the absence of implementation funds as a benefit, in recognition that additional finances are not always needed to address problems, and that funds can also be found within existing budgets, leveraged from other existing projects or lobbied from other development partners, particularly in Uganda and Malawi:

What we’ve actually learnt with the help of PERFORM2Scale [project] is that even the little resources that we have, we should be able to plan, … so that the activities or the objectives that you want to meet can be met without actually saying that ‘no, we didn’t do this because we didn’t have adequate resources’. (DHMT member, Malawi)

#### Improved teamwork

As a result of the structure of the MSI cycle, DHMTs in all countries held more frequent meetings with more DHMT members attending. They valued these meetings where they can interact with DHMT members who they do not regularly meet, learn from each other about how they manage their work and tackle problems, as well as discuss challenges and jointly problem solve.

Since the introduction of the MSI tool, I have now come to realise that you can’t do it alone, you need the collaborative efforts of your colleagues [in the DHMT]. You need their ideas, their suggestions then together we move forward. You can’t do it alone. (DHMT member, Ghana)

#### Gender considerations

The problem analysis and strategy development components supported the DHMTs to more readily consider gender, which had not previously featured as part of how DHMTs thought about problems or designed solutions. As a DHMT member from Uganda explained, ‘we used to not bother to look at gender but now after the MSI workshops, we consider gender while posting new staff’.

#### Factors affecting management strengthening

##### Multiple cycles

Going through multiple cycles of the MSI supported DHMTs to deepen their learning about what works in their settings and to learn from other DHMTs. For example, the first district group in Uganda went through three MSI cycles, which cemented their skills that would enable them to continue the MSI.

There are certain activities that we can surely carry on, for example, given the training the MSI cycles itself has given us, problem identification, prioritization, these are things that we are going to continue in our system. As managers, we are going to face problems and we need to find strategies. So, the knowledge is going to remain. (DHMT member, Uganda)

##### Research team role

The work of the research teams was a significant facilitating factor in scaling-up the MSI. They performed the dual roles of researchers and offering implementation guidance to RTs and DHMTs, which required both knowledge and skill on AR, as well as a deep understanding of the context, stakeholders and power relations. Without this type of implementation soft skills, progress with both horizontal and vertical scale-up would not have been possible.

### 4: Country scale-up strategies were developed

All countries drafted scale-up strategies. The initial strategy included the establishment of the scale-up structures and the horizontal scale-up plan for the project (see sections 1 and 2) and was developed together with the NSSGs and RTs in 2018. The initial strategy was then further developed over the next few years to include what happens beyond the initial horizontal scale-up supported by the project, and incorporates the concepts of handover and funded absorption of the MSI into existing structures and policies. This process was largely driven by the research teams, instead of the NSSGs and RTs, because of the limited functioning of the NSSGs. The research teams held discussions with the NSSGs or RTs in all countries which centred on: the vision for scale-up of the MSI over the next 5 years; adaptations to the MSI; strengthening of the steering group and plans for embedding the MSI into existing structures, policies and plans; working with stakeholders and champions for scale-up support; resources required; and monitoring and evaluation. While the intention was to further develop the scale-up strategy with NSSG members from all three countries during a March 2020 workshop, the consortium was unable to meet in-person due to COVID-19. Virtual meetings continued, but these were hampered by the NSSG and RT members having to prioritize the COVID-19 response and the limited functioning of the NSSGs. Ultimately, draft strategies included some of the elements listed above, but omitted monitoring mechanisms, milestones or indicators, and had limited stakeholder engagement and advocacy plans.

In Ghana, the development of the vertical scale-up strategy took time, with suggestions of establishing a regional-level version of the NSSG, and the integration of the MSI into regular DHMT refresher training and regional health authority routine support activities both featuring as part of the draft strategy. The strategy has yet to be validated by the NSSG and other stakeholders, however, and will need to be approved by the Director General of the Ghana Health Services and the MoH.

In Malawi, the vertical scale-up strategy was drafted by the RT, Quality Management Directorate (QMD) of the MoH and the research team, with the NSSG showing commitment to adopting the document. The presence of MSI champions within the QMD led to the human resources, health financing and gender information elements of the PERFORM2Scale project situation analysis tool being integrated into the nationwide Integrated Supportive Supervision tool. The satellite offices of the QMD were proposed as hubs for the scale-up of the MSI, with the offices’ quarterly review meetings accommodating MSI workshops and inter-district meetings.

In Uganda, the NSSG-FP, RT and research team worked collaboratively to develop the scale-up strategy. Early discussions about the MSI and its similarities with the existing quality improvement cycle resulted in engagement with the Quality Assurance and Improvement Department. The scale-up strategy describes the existing quality improvement and assurance structures providing governance oversight at the national level as well as the Quality Improvement Teams and Community Health Departments within the 14 regional referral hospitals supporting implementation. The human resource management focus of the MSI is included in the nationwide Quality Improvement (QI) strategic plan and framework (Ministry of Health Uganda, 2021).

#### Factors affecting the development of the scale-up strategies

##### Clarity and vision

There was a lack of clarity amongst the NSSG and RT in what the scale-up strategy should look like. While guidance was provided, the guidance intentionally avoided being over-prescriptive to instead encourage country ownership of the process and output. A clear, shared vision among the different stakeholders about how to integrate (components of) the intervention into existing systems is a critical part of the strategy and it took time to develop this shared vision.

##### Lost momentum with COVID-19 emergence

Just as ideas for scale-up were beginning to emerge in early 2020, the work itself was put on hold due to COVID-19. Unfortunately, we never regained the opportunity to meet face-to-face as a consortium and to share knowledge about scale-up experiences. Though we did make good use of webinars and workshops for communication, this was very much a second-best option for creatively developing and validating country scale-up strategies.

Factors affecting the functioning of the NSSG (section 1a) and few champions for scaling up the MSI (Section 5) also played an important role in the development of the scale-up strategies.

### 5. Some integration of MSI into policies and practice in Uganda and Malawi, but no financial support

While the funding for scale-up is not yet in place in all settings, strong indications of scale-up are present in Malawi and Uganda. In Malawi, the scale-up strategy has the backing of senior members of the MoH, but its successful implementation depends on how well the MSI workshops can be integrated into the satellite structure and quarterly review meetings, in addition to the financial support needed to make this happen. Implementation of the scale-up strategy will also depend on the functionality and acceptability of the QMD satellite offices. Before integration of the MSI can take place, more clarity on the roles and responsibilities of satellite offices towards DHMTs and other sectors is needed.

In Uganda, integration of the human resource management focus of the MSI into the QI framework brings several opportunities. The regional QI teams will take a leading role in implementing the QI cycles, but only a few of the 14 regional-level teams are currently active and appropriately skilled to facilitate these cycles. Therefore, the NSSG-FP stressed that the scale-up strategy should include strengthening capacity in regional QI teams, with the intention being that these teams will eventually take on a role similar to a regional RT. Adequate financial support for the new QI framework also remains an issue. There will be some government budget for full implementation of the QI strategy in all regions, but additional resources from development partners may be needed.

In Ghana, there is currently no evidence of political and financial support for the scale-up of the MSI and this is due to challenges in engaging with relevant national-level stakeholders. There are currently no concrete plans to integrate the MSI into a policy document, budget, training curriculum or guidelines in Ghana, but discussions are ongoing.

#### Factors affecting integration

##### Champions for scaling-up the MSI

Strong supporters of the MSI did emerge, but only from among those who were close to the PERFORM2Scale project. These supporters played an important role in advocating for the MSI. For example, in Ghana, champions included DHMT members exposed to the MSI and regional health directors. These champions advocated for the scale-up of the MSI at a small scale but not at national level, mostly through sharing experiences of the MSI. In Malawi, some RT members, heads of the DHMTs of well-performing districts and members of the QMD were all identified as champions. The relatively new QMD saw supporting PERFORM2Scale project as an opportunity to implement a novel approach suited to their remit. In Uganda, the NSSG-FP emerged as a strong champion of the scale-up process and was strategically placed to inform and guide the scale-up given his previous engagement as District Health Officer in the PERFORM project and his experience across high-level positions in the MoH.

PERFORM2Scale project had initially intended for each country to hold annual national workshops, where a wide group of stakeholders could discuss the successes, challenges and lessons learned in the scale-up process. Despite attempts, bringing high-level decision-makers together amidst busy schedules proved too challenging. More champions may have emerged if these workshops had taken place more regularly.

##### Generating and sharing robust quantitative evidence and costing data early

Although we were able to provide compelling narratives on improvements in management, health workforce and service delivery, we lacked robust quantitative outcome data to support these narratives. Given the preference for quantitative data, including costing data, among key stakeholders in all countries, gaining stakeholder support was challenging in the absence of being able to demonstrate quantifiable benefits. Presenting the MSI as a low-cost intervention early on might have been attractive to government stakeholders and donors. When these data became available, the cost of one MSI cycle for one district was deemed to be relatively low, at an average of $26 000 (PERFORM2Scale, 2022).

The other challenge [..] is that evidence would be appreciated more if it was quantitative. And every time they asked us for evidence, they wanted to see numbers. But the issue which we learnt over time is that management is a bit complex in a way that it does not necessarily always give you numbers, it only gives you proxies and what matters in management is mainly the processes that happen around that actually contribute to the service delivery. (Research team, Uganda)

#### Power and politics

Although evidence played a role in convincing stakeholders of the value of the MSI scale-up, it also depended on their position and mandate. In Ghana, gaining buy-in of the Regional Director of Health Services in the early part of the MSI scale-up facilitated horizontal scale-up across the region. However, national-level stakeholders relevant for the scale-up were not yet fully convinced of the value of the MSI. Evidence supporting scale-up was available but despite numerous attempts to get the PERFORM2Scale project MSI on the agendas of national fora (e.g. the Annual Health Summit) this has not yet materialized.

We have all agreed that we have gathered enough evidence to support the scale-up of the MSI in other districts but then we still have a few more steps to go, like what we have just discussed, to talk to the major stakeholders involved with the scaling-up of the MSI. (RT member, Ghana)

In Malawi, the MSI was aligned with national political interests in improved district-level leadership. The QMD was convinced of the value of the MSI scale-up and as its director was well-respected within the MoH, he was able to steer the scale-up with little involvement of the Senior Management Team. However, to get further support, which is essential for the MSI to be funded and implemented, more information needs to be provided to wider groups of stakeholders. Meetings with UNICEF have taken place, which provides a platform for further discussion about financing the MSI.

In Uganda, it was clear that not all stakeholders were on board with the scale-up of the MSI. It took time, evidence and discussion to change people’s views on the MSI as described below:

The current (Human Resources) commissioner who has come on board is a very senior person, has worked in the sector for very long and has worked in different ministries and they have tried so many approaches to improve workforce performance, maybe sometimes without success. So he came on board with that belief that it’s not possible, but we managed to sit him down and have one [meeting] with him, to give him the evidence available and in my view, his view is changing. (NSSG, Uganda)

## Discussion

The purpose of this paper was to describe the process and associated outcomes of scaling-up the MSI across all three PERFORM2Scale project countries and through this generate new knowledge that can be used by other health practitioners, decision-makers and researchers seeking to scale-up a complex health intervention.

Overall, our results show that horizontal scale-up of the MSI was facilitated by the repeated use of MSI cycles, which, over time, improved DHMTs’ confidence and independence in problem solving and strategy development. This was evidenced by more creative use of existing resources, improved teamwork and, albeit to a lesser extent, the consideration of gender within the problem identification stage. This finding adds to previous findings generated during PERFORM (Martineau *et al*., 2018), suggesting that the MSI remains an effective intervention for management strengthening. The opportunity to carry out multiple cycles of the MSI within PERFORM2Scale project further evidenced how the MSI could be implemented without additional funds for implementation of strategies—seen by many as a positive attribute—with DHMTs reporting that the MSI contributed to improvements in workforce performance and service delivery. The combined efforts of the research teams and the RTs worked to improve the confidence and autonomy of DHMTs to implement the MSI, as key contributing factors to the successful horizontal scale-up of the MSI across a total of 27 districts, across three countries, over 4 years. In terms of vertical scale-up, there was some integration of the MSI into policies and practice in Uganda and Malawi but not in Ghana.

From these findings, we have identified the following critical lessons about scaling-up complex health interventions, which we discuss in relation to other literature.

### Arguing the value of the intervention

Evidence is needed to convince stakeholders about scaling-up an intervention. In this study, we found that the MSI concept was not easy to sell to wider stakeholders or to get support from champions. The impact of management strengthening is difficult to demonstrate, as its effects may not be immediately observable (Horton *et al*., 2000). Also, unlike simpler health interventions, it was difficult to demonstrate the direct impact of the MSI on service delivery, with quantitative evidence and costing data not available at opportune times to demonstrate value-for-money. Major efforts are therefore needed to generate timely evidence and to disseminate this evidence in a way that is acceptable and important for decision-makers.

Gaps between the end of the PERFORM project in 2014, the development of the proposal for the PERFORM2Scale project in 2016 and its implementation in 2018 meant that the benefits of the MSI under PERFORM may have been lost to staff turnover and other factors. A smoother transition from pilot study to scale-up needs to be considered, in line with ExpandNet’s mantra of ‘begin[ning] with the end in mind’ (World Health Organization /ExpandNet, 2011).

### Building a coalition and the structures for scale-up

While the original assumption was that NSSG members needed to be high-level managers to enable decision-making, their unavailability limited their meaningful involvement. Therefore, the use of more readily available structures, such as the Technical Working Groups in Uganda and RSSGs in Ghana, in combination with intervention champions, is advisable for scale-up efforts going forward. Scale-up was facilitated using RTs, as a more stable and accessible structure than the NSSGs, allowing for sustained institutional memory of the MSI, better information sharing across district groups and the emergence of MSI champions. This was considered particularly important given that the MSI concept was not easy to ‘sell’ to wider stakeholders, possibly as no specific funding was attached to the intervention. Ultimately, MSI champions proved indispensable to understanding stakeholders’ interests, relationships and networks, local power dynamics and influences. A clearer ‘picture’ of what a champion is and what they do, including the difference between being convinced and supporting the intervention vs actively lobbying for scale-up, would allow for the identification and more effective use of champions going forward (Santos *et al*., 2022).

### Looking for windows of opportunity

Finding robust structures within which to incorporate the MSI was challenging. Alignment of the intervention to existing policies and interests should therefore be considered at the outset to form a clear, shared vision of scaling-up a complex health intervention (World Health Organization /ExpandNet, 2010). This requires both in-depth knowledge of the policy environment and building strong relationships with key decision-makers, which, in rapidly changing (i.e. decentralizing) contexts, could be facilitated via champions identified through the regular use of stakeholder analyses or other methods such as political economy analysis (Serrat, 2017). While every effort was made to accomplish this (i.e. via an evaluation of PERFORM which included discussions with stakeholders about next steps; government support letters for the PERFORM2Scale project proposal; initial context analysis interviews; and reviews with Ghana and Uganda DHMTs in the first year), the research team failed to pick up on key factors, including the development of the QI strategy in Uganda; the evolution of decentralization in Malawi; and the potential impact of focusing initial scale-up in one region in Ghana, rendering national engagement more challenging.

### Realizing the need for trade-offs in changing contexts

In addition to the COVID-19 pandemic, we found that during the intervening period from proposal writing in 2016 to start of project implementation in 2018, there were other key changes in the country settings. It is thus important to recognize that there is a trade-off between maintaining the integrity of an intervention and the need for its adaptation to changing circumstances (Chambers and Norton, 2016; Bulthuis *et al*., 2020; Kirk *et al*., 2020). Our study has also highlighted the need for dynamic scale-up processes that respond to changing contexts. For example, in Uganda we responded to the opportunity of contributing the integration of the human resource management focus of the MSI into the QI framework being developed by the Ministry of Health.

### Financing scale-up

Scale-up of the MSI was initiated from ‘outside’ with external funding provided as part of the research project, rather than funding sourced from national resources or prioritized by national government using donor resources. The absence of earmarked funds for the scale-up beyond the PERFORM2Scale project in all the countries raises the question of whether one should embark on a scale-up process without first carrying out an estimate of ongoing running costs to support the scale-up and the assurance that future funding would be available. Or indeed whether it is better to generate support for scale-up as you go along, as proposed by ExpandNet (World Health Organization /ExpandNet, 2010). Perhaps a combination of both approaches is needed.

### Thinking and working politically

Underlying all of these lessons is thinking and working politically throughout the scale-up process: identifying who we should work with—based on interest, influence and the power to make decisions and influence others; understanding how to leverage the position, influence and networks of champions so they not only support the MSI but actively advocate for its scale-up, was critical; and anticipating (changes in) power relationships between key stakeholders and decision-makers. Identifying emerging stakeholders as contexts are changing was important, e.g. during decentralization process and new governments as we saw in Malawi. Guidance on existing tools available and formal approaches for political economy analysis are discussed by Whaites (2017). Furthermore, scale-up is a dynamic and non-linear process that requires constant assessment of the context and adaptation of the scale-up approach.

## Strengths and weaknesses

The strengths of this study lie with the experienced and embedded research teams present in each context who supported one another through the research process. Our collective perspectives enriched the data collection, analysis and interpretation across diverse contexts to incorporate multiple settings and voices in support of the broader generalizability of the findings. The combination of process evaluation with outcome evaluation is a strength. The study, however, is not without limitations. First, the impact of management strengthening, as the purpose of scaling-up the MSI, is difficult to demonstrate as effects can take time to observe. Unlike less complex or clinical interventions, it was difficult to demonstrate the direct impact of the MSI on service delivery, with quantitative evidence and costing data not available at opportune times to demonstrate value-for-money. This challenge illustrates the need for conceptual models and theories of change to guide the scale-up process. Second, while we used a range of methods to tell the story of the scale-up of the MSI, this was not an independent evaluation whereby researchers supported both the implementation and scale-up of the MSI and the evaluation of its effects. To limit this potential bias, research teams discussed their positionality, and used online platforms to critique their findings and interpretations across the contexts.

## Conclusion

PERFORM2Scale project successfully scaled-up the MSI to 27 districts across 3 countries. Scale-up strategies were developed in each country with integration of the MSI into policies and routine practice in Uganda and Malawi, all in the absence of additional financial support. In Ghana, the scale-up strategy has not yet been integrated into policies or routine practice. Through these experiences, we have identified key factors that are likely to contribute to successful scale-up. Working with champions who are well-positioned within existing political structures and who can readily identify current practices and policies that would benefit from the integration of an intervention is critical. As too is engaging in regular and consistent political and stakeholder analyses, in order to monitor changes in rapidly evolving systems. Considering how interventions might be better communicated and marketed to suit existing priorities and policies is essential, even if this may result in substantially modified, albeit more contextually appropriate, interventions. The importance of securing funding commitment for whatever form the intervention eventually takes is needed to ensure its longer-term sustainability.

## Supplementary Material

czae063_Supp

## Data Availability

The data that support the findings of this study are available from the corresponding author upon reasonable request.

## Abbreviations

AR = Action research

DG = District Group

DHMT = District Health Management Team

MoLoGRD = Ministry of Local Government and Rural Development

MOH = Ministry of Health

MSI = Management Strengthening Intervention

NSSG = National Scale-up Steering Group

RT = Resource Team

WHO = World Health Organization

UHC = Universal Health Coverage

## Reflexivity statement

The authors are a diverse group of women and men of different ages, seniority, nationalities and ethnicities. The authors include six women and five men, four are African, five are European and one is Canadian. The authors are at different levels of seniority from postgraduate researchers to professors in global health.
