# Supplementary material for: From PERFORM to PERFORM2Scale: lessons from scaling-up a health management strengthening intervention to support Universal Health Coverage in three African countries
Source: Health Policy Plan. 2024 Jul 9;39(8):841–53. doi: 10.1093/heapol/czae063 (PMC11384104; doi:10.1093/heapol/czae063)
Supplement: czae063_Supp [file czae063_supp.zip › Supplementary File 1 Data collection tools.docx]

# In depth interview guide

- - - 1. **Can you please introduce yourself?**
- What is your role within the DHMT- For how long do you hold this role?
- How and since when are/were you involved in the Perform2Scale management strengthening intervention (MSI)?

1. **After finishing the MSI cycle(s), which changes did you notice?**

- Changes at **individual/personal level** (probe and relate to workplan: better personal competencies, more confidence, more clarity/better understanding on responsibilities/level of decision space)? Examples
- Changes in **management skills and team work within the DHMT**? Examples? For example how about trust for collaborations, better interactions among different members of the team, units, empowered to express the opinion freely? Were the skills that have been developed during the MSI useful for addressing COVID? If yes, which of them? Give example
- Changes in **organisational practices and procedures** (probe and relate to workplan: time management, prioritising problems, team management, efficient resource usage)? Examples?
- Changes in **workforce performance** such as motivation and absences of staff (probe and relate to workplan: supportive supervision, a collaborative workplace, discussion for decision-making, improved competencies, incentives, less turnover, HWs attitude, attendance in workplace)?  Examples?
- How did the MSI approach influence the work performance?
- Were health workers better motivated respectively could you observe changes in work performance? If yes, why? If not, why?
- Did the MSI approach influence the level of work attendance and absences?
- Did the MSI approach make the workplace more attractive? If yes, how? If not, why not?
- Changes in **service delivery problems** within the district, such as for example quality of care and access to services. In other words, how did the workplan implementation impact the service delivery within the district, in terms of quality of care, access to services?
- Changes in **considerations regarding gender equity** within the team? Why so? Did you consider different gender-based approaches while implementing the workplans?

1. **In your opinion how long does it take until the DHMT can make full use of the MSI approach?**

- Did you have the necessary time for using the MSI approach? If not why not? What would need to change for you been given the required work time for using the MSI approach?
- Looking back what do you consider to be the essential skills for implementing the MSI approach?
- When can the DHMT manage the MSI process themselves, without facilitation?
- How many supervisory visits from the CRT/RT did you get and what was their impact? Were these visits helpful?

1. **In your opinion, has the MSI approach produced the expected results and were these results adequately available and disseminated?**

- What are the key results and outcomes achieved because of the MSI approach in your district?
- Were these outcomes appropriately documented and disseminated? If yes, please explain (and where?). If not please indicate what is to be improved

1. **Did the MSI-approach become integrated in the regular health planning processes in your district? If so, how?**

- What were the consequences?
- If not why MSI-approach did not become integral part of the regular health planning processes and what were the consequences?

1. **Are there “champions” advocating for the MSI approach or scale-up of the MSI approach?**

- Who are these champions?
- If yes, can you give examples of what they did?
- If not, why not?

1. **What do other stakeholders think about the MSI approach?** *(Stakeholders such as national level, local government, population etc.)*

- Did the MSI approach lead to new joint activities/partnerships?
- Do you discuss about MSI during meetings with other actors?
- Are they convinced of the value of the MSI? Why? /not?
- Which other stakeholders would you like to see involved in the MSI and why? How would you invite them to join? How could other stakeholders or DHMTs be reached and convinced on the value the MSI approach?

1. **Has the MSI approach been used to address other health system dimensions/programmes** (improvement of availability of drugs, supplies, maintenance of equipment, quality of care)?

# Scale-up assessment tool

**PHASE 1**

**Indicate the extent to which you agree with the following statements:**

|  | **Strongly disagree** | **Disagree** | **Neutral** | **Agree** | **Strongly agree** |
| --- | --- | --- | --- | --- | --- |
| 1. All necessary stakeholders for scale-up of the MSI are convinced of the value of MSI by the available evidence. |  |  |  |  |  |
| 1. A contextually appropriate scale up strategy is used to scale up the MSI |  |  |  |  |  |
| 1. There are people (champions) advocating for the scale-up of MSI |  |  |  |  |  |
| 1. The RT includes the right people with the right capacity to implement the scale-up the MSI (horizontal scale-up). |  |  |  |  |  |
| 1. The NSSG includes the right people with the right capacity to steer the scale-up of the MSI |  |  |  |  |  |
| 1. The decision makers show political and financial support to scaling-up the MSI |  |  |  |  |  |
| 1. The monitoring of the scale-up of the MSI is used to adjust the scaling-up process |  |  |  |  |  |

**PHASE 2:**
After the first phase, collect the results of the statements and discuss with the participants why they have scored like this. Ask for example why a lot of people agreed with statement X. Use the prompts of the guided discussion guide to further understand the perceptions of the participants. It is important to ensure that everyone participates during the guided discussion.

| **Statements** | **Prompts** |
| --- | --- |
| 1. All necessary stakeholders for scale-up of the MSI are convinced of the value of MSI by the available evidence. | General note on specification of stakeholders: intentionally left vague/open ended but to be analysed using the six stakeholder interactions outlined by Campos & Reich (interest, bureaucratic, budget, leadership, external, beneficiaries)   - Who are these stakeholders? - What is their interest in the MSI (see categories above for probing) and how influential are they? - Why are they convinced/not convinced? - How do we ensure that key stakeholders will be convinced/see the value of the MSI? - Which evidence is available? Qualitative and quantitative? Experiences? How is this evidence used? - Are you convinced of the value of the MSI? How do you see the balance between the impact/results vs. the resources used. |
| 1. A contextually appropriate scale up strategy is used to scale up the MSI | - How was the strategy developed or evolved? - Who is involved in the strategy development? - How is the scale-up strategy used? - What could be improved in the process of strategy development **and** content of strategy? - What needs to change in the scale-up strategy in the context of COVID? - What needs to change in the scale-up strategy to ensure continuation of the scale-up process after this programme? - How are the different stakeholders working together in implementing the scale-up strategy? - What are key challenges in working together to implement this strategy? Or in getting these stakeholders involved? and opportunities? (probe for strained/interactions between the six stakeholder groups) - Do you think that scale-up will continue after this project? Why/why not? |
| 1. There are people (champions) advocating for the scale-up of MSI | - Who are these people advocating for the scale up of the MSI? - At which level of the system are they advocating? - What is their reason/personal motivation to advocate for the scale-up of the MSI? - How do they advocate for the scale-up of the MSI? - What are the results of people advocating for the scale-up of the MSI? (political, financial support?) - How did these people advocating for the MSI emerge? - Is there an advocacy strategy developed as part of the scale-up strategy? - Are the right people advocating for the MSI to make a difference? What makes them the right people (skills/background etc.)? |
| 1. The RT includes the right people with the right capacity to implement the scale-up the MSI (horizontal scale-up). | - Who are the right people, why and who is missing and why? - Is the RT (as a body) ‘fit for purpose’ to implement the horizontal scale up of the MSI? - Who decided who is in the RT and how members are replaced/new members will join? - What are the roles of the RT? Are these clear to CRT, to RT and to NSSG? - Barriers or facilitators to well-functioning of the RT?   - Sufficient time for the RT to implement?   - What capacity is needed for the RT and what capacity is still missing? (communications, facilitation, - Is the RT committed/pro-active? Any difference among the different RT members? - Involvement in developing a scale up strategy and involvement in vertical scale-up process? - How are the role divisions within the RT team? - How is the relationship between the RT/NSSG? How are they collaborating? - What is your perception on the sustainability of the RT? |
| 1. The NSSG includes the right people with the right capacity to steer the scale-up of the MSI | - Who are the NSSG members? - Who are the right people and who is missing? - Is the NSSG (as a body) ‘fit for purpose’ to steer the scale-up up of the MSI? - Who decides who is in the NSSG and how members are replaced/new members will join? Did the position of people play a role in the selection of NSSG members? - Do they take the leadership in the scale-up of the MSI? - Are the main influencers of decision making/decision makers on scale-up of the MSI part of the NSSG? - Is the NSSG committed/pro-active in fulfilling the role that they have? - Are the roles of the NSSG clear? /What do you expect each member of the NSSG to do for the scale-up? Is this done? Why or why not? - Barriers or facilitators to well-functioning of the NSSG?   - Sufficient time of the NSSG to steer scale-up?   - Does the NSSG have sufficient overview/contextual information to steer scale up of the MSI? What’s lacking?   - What capacity is needed for the NSSG/ what capacity is still missing? (communications, facilitation, negotiation, partnership formation and management, to conduct the scale-up of the MSI) - What is your perception on the sustainability of the NSSG? |
| 1. The decision makers show political and financial support to scaling-up the MSI | - Who makes the decision about the scale-up of the MSI? - What does show political support mean? (passive political support vs. active leadership?) - What does show financial support mean? - What are barriers/opportunities to secure this support? - Who should pay for the scale-up of the MSI? Why? - Did you use insights in the cost-benefits of the MSI to convince the decision maker? |
| 1. The monitoring of the scale-up of the MSI is used to adjust the scaling-up process | Separate questions for horizontal scale-up and vertical scale-up.   - How does monitoring take place, how often, who is doing it and how do findings get translated into action (what kind of action?)? What type of information is collected? Is this the info that is needed to improve scale up? Why or why not? - How can monitoring of the scale-up be improved? - Provide examples of changes introduced based on the monitoring system. |
| **IF TIME ALLOWS DISCUSS THE FOLLOWING STATEMENTS** | |
| 1. The benefits of the MSI process justify the resources used for the implementation of the MSI process (from situation analysis to implementation and which mainly involves external resources for funding the workshops etc., and RT/DHMT members’ time) | - Do you know what resources are used? - If yes, how many resources are used for implementation of MSI? - What are the benefits of the MSI? - How is the balance between the impact/results vs. the resources used. - Potential adaptations which may be needed to facilitate the scale-up of the MSI and/or sustainability of the MSI. |
| The DHMTs are well capacitated on the MSI approach | Note: DHMTs can pursue MSI approach at least on a yearly basis on their own vs. DHMTs do use MSI approach at least on a yearly basis on their own   - In the context of the MSI, what do you think it means to be well capacitated” - Why are they capacitated or why not? Capacity of DHMTs (baseline) or the process of building capacity? - Has the MSI altered power and gender dynamics within DHMTs? In what way? - How could the capacity of the DHMT be improved? |

# Reflection with research team tool

| **Questions** |
| --- |
| **Scale-up** |
| 1. What is going well during the scale-up of the MSI? Why? |
| 1. What is going not so well during the scale-up of the MSI? Why? |
| 1. Are all the necessary stakeholders convinced of the value of MSI by the available evidence? Why or why not?  - Who are these key stakeholders? - What is their interest in the MSI? |
| 1. Are the DHMTs well capacitated on the MSI approach? Why or why not? |
| 1. Is a contextual appropriate scale-up strategy used for the scale-up of the MSI? Why or why not?  - Reflections about the development of the scale-up strategy - Reflections about the content of the scale-up strategy - Reflections about the governance structure (NSSG/RT) – was/is this suitable for the specific country context? |
| 1. Are the necessary stakeholders involved in scaling-up the MSI working in partnership? Explain and provide examples.  - Who are the necessary stakeholders? - What are the challenges in working together/getting them involved? |
| 1. Are there champions advocating for the scale-up of MSI?  - Who are they? At which level of the system are they advocating? - How do they advocate? - What are the results of them advocating? |
| 1. Does the RT include the right people with the right capacity to implement the scale-up the MSI? Why or why not?  - Who decides who is in? - Fit for purpose to implement the horizontal scale-up? Committed/pro-active? - Barriers/facilitators? - Relationship between NSSG/RT? - Sustainability of RT? |
| 1. Does the NSSG include the right people with the right capacity to steer the scale-up the MSI? Why or why not?  - Who decides who is in? - Fit for purpose to steer the scale-up of the MSI? Committed/pro-active? - Barriers/facilitators? - Relationship between NSSG/RT? - Sustainability of NSSG? |
| 1. Show the decision makers (at national level) political and financial support to scale-up the MSI? Why or why not?  - Who are the decision makers? - How does political/ financial support look like? - Barriers/facilitators? |
| 1. Is the monitoring of the scale-up of the MSI used to adjust the scaling-up process? Why or why not?   Provide examples of adjustments done or needed based on monitoring data. |
| **MSI** |
| 1. What went well during the implementation of the MSI? |
| 1. Why are these (the above mentioned) things going well? |
| 1. What is not going well during the implementation of the MSI? |
| 1. Why are these (the above mentioned) things not going well? |
| 1. Did you notice any differences in the MSI implementation when the RT implemented the MSI compared to when the CRT did this/supported? (only relevant for Ghana and Malawi). |
| 1. What do you think of the feasibility of continuing the MSI approach post project/when CRT stops supporting the process? |
| 1. How could the implementation of MSI be improved?  - Adaptations necessary to facilitate vertical scale-up? - Adaptation necessary to ensure sustainability of the MSI? |
| 1. How has the implementation of MSI contributed to more attention of the DHMTs to equity and gender? How could this be improved? |
| **Role of CRT** |
| 1. How has the CRT influenced the implementation of the MSI and the scale-up? (as CRTs are wearing multiple hats) |
| 1. What are the challenges that the CRT has faced in facilitating the MSI and scaling it up? How were they or should they be overcome? |
| 1. How has the CRT influenced the research process regarding the MSI and scale-up of the MSI? (as CRTs are wearing multiple hats) |
| 1. How have you as an individual influenced the implementation of the MSI and the scale-up (e.g. connections, relations etc.)? |
