# Supplementary material for: From PERFORM to PERFORM2Scale: lessons from scaling-up a health management strengthening intervention to support Universal Health Coverage in three African countries
Source: Health Policy Plan. 2024 Jul 9;39(8):841–53. doi: 10.1093/heapol/czae063 (PMC11384104; doi:10.1093/heapol/czae063)
Supplement: czae063_Supp [file czae063_supp.zip › Supplementary File 3 Location of study districts.docx]

**Supplementary file 3: Locations of participating districts by country**

**PERFORM2Scale districts in Ghana**


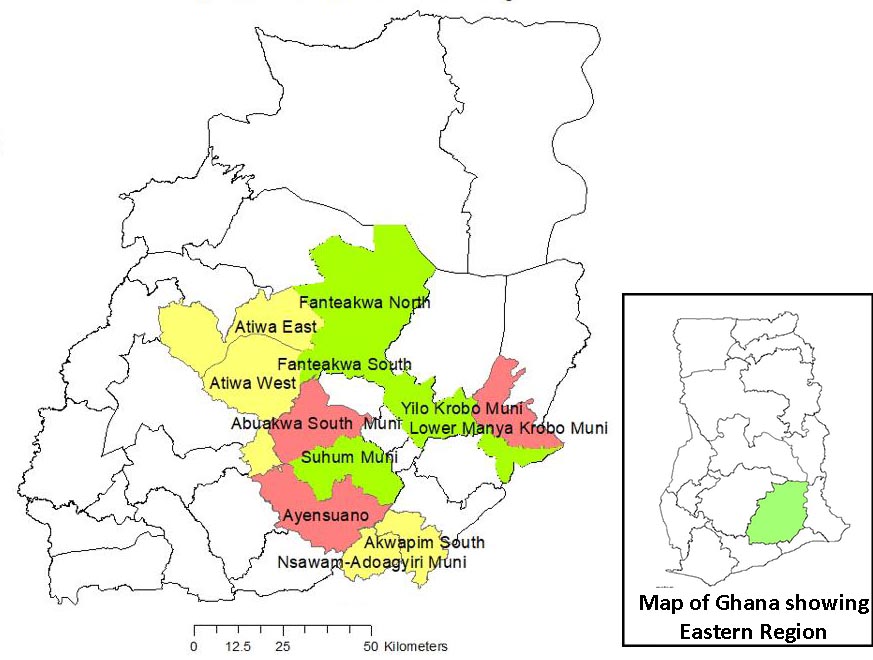


**DG1 - Fanteakwa, Yilo Krobo and Suhum**

**DG2 - Ayensuano, Lower Manya Krobo and East Akim**

**DG3 – Nsawam-Adoagyiri, Akuapim South and Atiwa**

**PERFORM2Scale districts in Malawi**


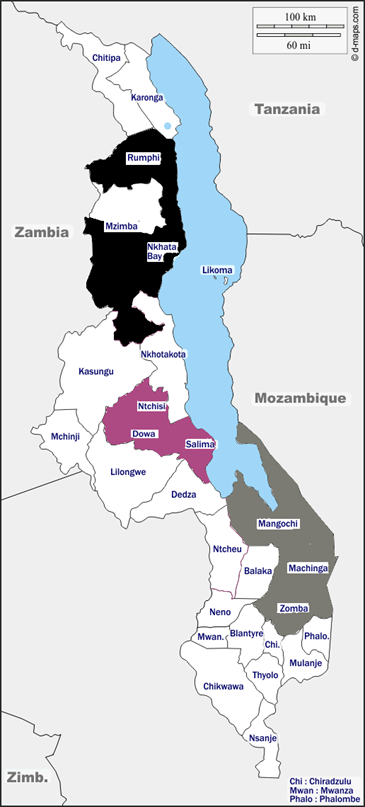


**DG1 - Dowa, Ntchisi and Salima**

**DG2 - Machinga, Mangochi and Zomba**

**DG3 - Mzimba south, Nkhata Bay and Rumphi**

**PERFORM2Scale districts in Uganda**


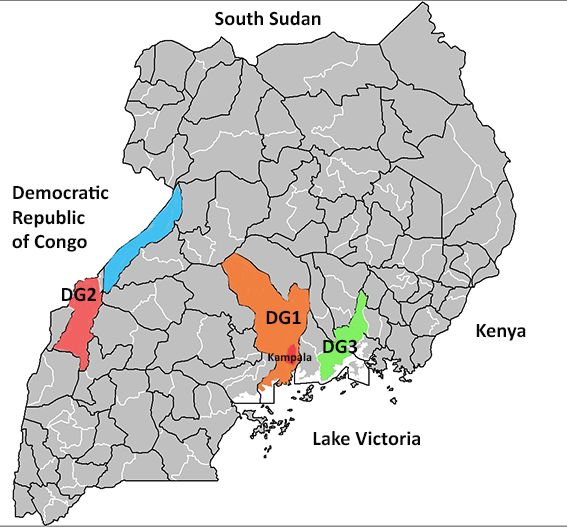


**DG1 - Luwero, Nakaseke and Wakiso**

**DG2 - Kabarole, Ntoroko and Bunyangabu**

**DG3 - Jinja, Luuka and Buikwe**
