# Supplementary figures and images for: From PERFORM to PERFORM2Scale: lessons from scaling-up a health management strengthening intervention to support Universal Health Coverage in three African countries
Source: Health Policy Plan. 2024 Jul 9;39(8):841–53. doi: 10.1093/heapol/czae063 (PMC11384104; doi:10.1093/heapol/czae063)

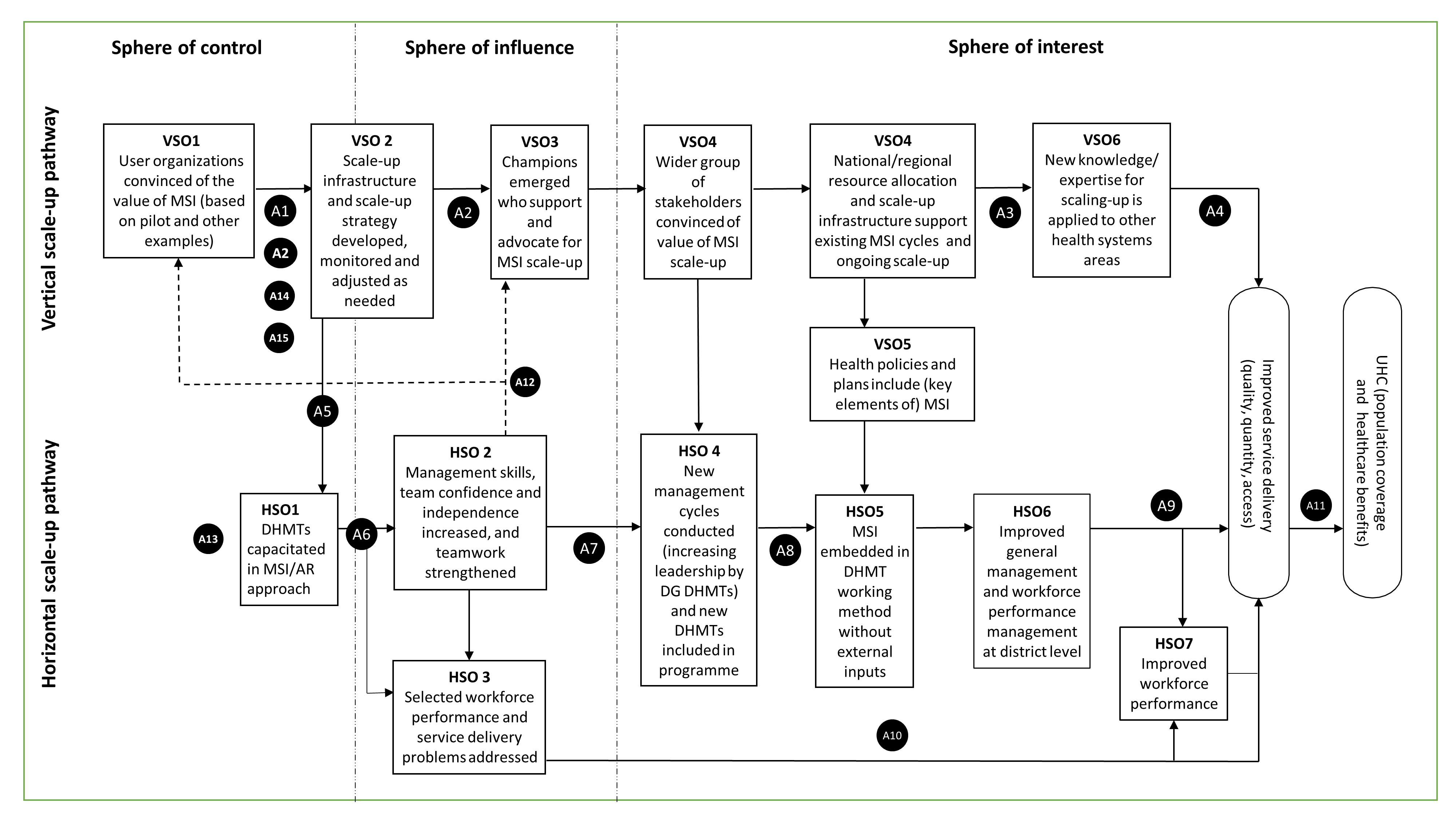

Supplement: czae063_Supp [file czae063_supp.zip › Supplementary file 2 Theory of Change.jpg]
